# Synthesis, Crystal Structure, Vibration Spectral, and DFT Studies of 4-Aminoantipyrine and Its Derivatives

**DOI:** 10.3390/molecules18010877

**Published:** 2013-01-11

**Authors:** Yi Li, Yuanyuan Liu, Haowei Wang, Xiaohui Xiong, Ping Wei, Fangshi Li

**Affiliations:** 1 College of Biotechnology and Pharmaceutical Engineering, Nanjing University of Technology, Nanjing 210009, China; 2 College of Food Science and Light Industry, Nanjing University of Technology, Nanjing 210009, China; 3 Department of Chemical and Pharmaceutical Engineering, Southeast University ChengXian College, Nanjing 210088, China; 4 College of Science, Nanjing University of Technology, Nanjing 210009, China

**Keywords:** 4-aminoantipyrine derivatives, X-ray structure determination, IR spectroscopy, DFT calculations, electronic structure properties

## Abstract

Three compounds derived from 4-aminoantipyrine (AA) were synthesized and their structures confirmed by melting point, elemental analysis, FT-IR, and ^1^H-NMR. The molecular structures of the four compounds were characterized by single-crystal X-ray diffraction and calculated by using the density functional theory (DFT) method with 6-31G (d) basis set. The calculated molecular geometries and the vibration frequencies of the AA derivatives in the ground state have been compared with the experimental data. The results show that the optimized geometries can reproduce well the crystal structural parameters, and the theoretical vibration frequencies show good agreement with the experimental data, although the experimental data are different from the theoretical ones due to the intermolecular forces. Besides, the molecular electrostatic potential (MEP) and the frontier molecular orbital (FMO) analysis of the compounds were investigated by theoretical calculations.

## 1. Introduction

**Figure 1:**
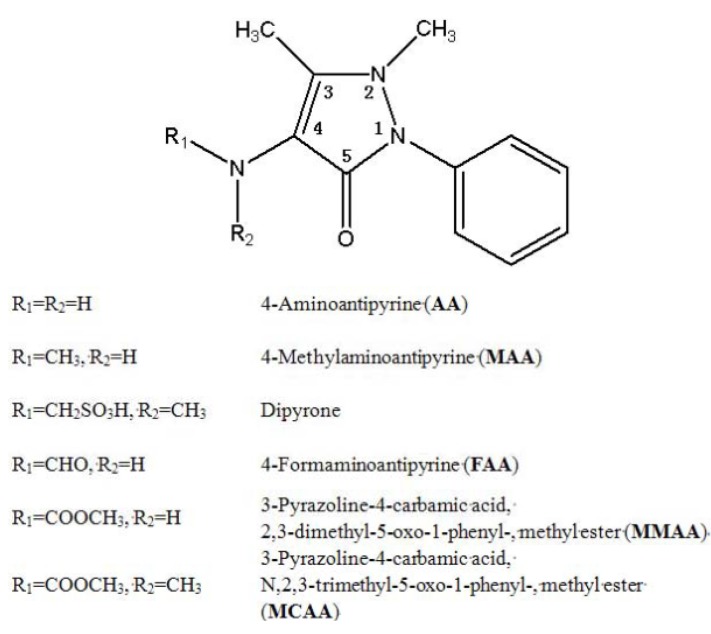
Structures of AA and some of its derivatives.

4-Aminoantipyrine (AA) and its derivatives (Figure 1) have potential biological activities [1,2,3,4,5,6,7], such as analgesic, anti-inflammatory, antimicrobial, and anticancer properties. Recently, AA and 4-methylantipyrine (MAA) were found to correlate with the analgesic effect of dipyrone [8]. A study demonstrated for the first time that dipyrone and some AA derivatives have a high potential to attenuate or prevent the anti-platelet effects of aspirin [9]. This was confirmed by docking studies, which revealed that MAA forms a strong hydrogen bond with serine 530 within the COX-1 enzyme, thereby preventing enzyme acetylation by aspirin. The three-dimensional structures of COX-1 and COX-2 have been solved by X-ray crystallography [10,11].

Although there have been many studies of the synthesis and biological activities of AA and its derivatives, there are only a few articles concerning the structures of these compounds. To our knowledge, there are no articles describing their complete structural analysis. Since AA and its derivatives are biologically active compounds, information about their 3-dimensional structures, especially their crystal structures, may be of great interest for rational drug design. On the other hand, we also aimed to obtain and analyze the electronic structures of AA and its derivatives. B3lyp theory with 6-31G* basis set was used since it is known to be quite a reliable method [12].

In this study, we present results of a detailed investigation of the structural characterization of AA and its three derivatives (FAA, MMAA, MCAA) using single crystal X-ray diffraction, IR spectroscopy, and quantum chemical methods. The geometrical parameters, fundamental frequencies of the three derivatives in the ground state have been calculated by using the DFT (B3LYP) method with 6-31G (d) basis set. This calculation is valuable for providing insight into molecular parameters and the vibration spectrum. The aim of this work was to explore the molecular dynamics and the structural parameters that govern the chemical behavior, and to compare predictions made from theory with experimental observations.

## 2. Results and Discussion

### 2.1. Crystallographic Results

**Figure 2:**
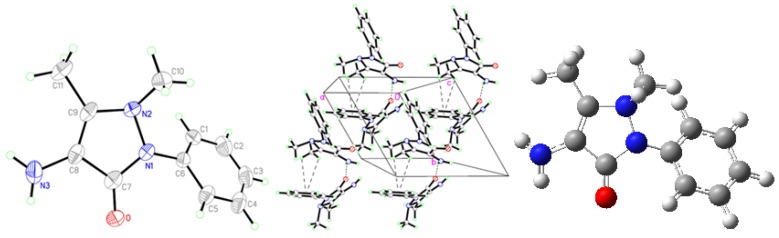
Crystal structure (**Left**), packing diagram (**Middle**), and theoretical optimized geometric structure (**Right**) of AA.

**Figure 3:**
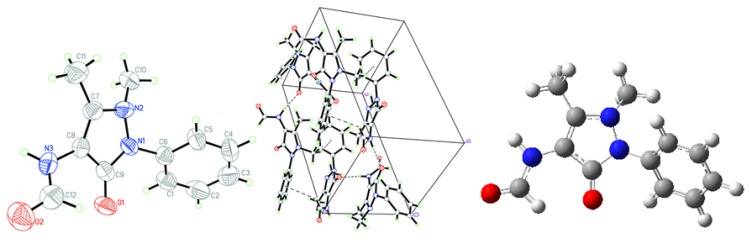
Crystal structure (**Left**), packing diagram (**Middle**), and theoretical optimized geometric structure (**Right**) of FAA.

**Figure 4:**
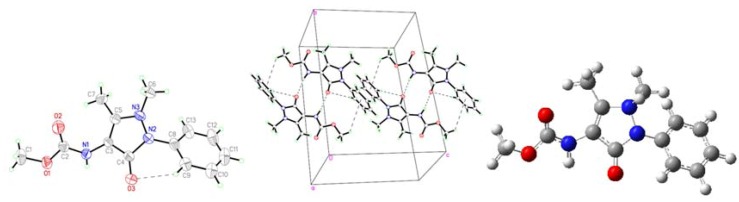
Crystal structure (**Left**), packing diagram (**Middle**), and theoretical optimized geometric structure (**Right**) of MMAA.

**Figure 5:**
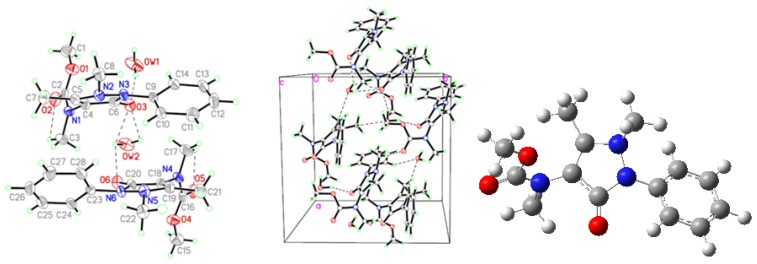
Crystal structure (**Left**), packing diagram (**Middle**), and theoretical optimized geometric structure (**Right**) of MCAA.

Crystal data of AA, MMAA, and MCAA are listed in Table 1. The selected molecular structure parameters (bond lengths and bond angles) are listed in Table 2, Table 3, Table 4, Table 5. The hydrogen bonds are listed in Table 6. The molecular structures and the packing diagrams are shown in Figure 2, Figure 3, Figure 4, Figure 5.

**Table 1:** Crystal and structure refinement data.

	AA	MMAA	MCAA
empirical formula	C_11_H_13_N_3_O	C_13_H_15_N_3_O_3_	C_28_H_38_N_6_O_8_
formula weight	203.24	261.28	586.64
temperature [K]	293 (2)	293 (2)	293 (2)
wavelength [Å]	0.71073	0.71073	0.71073
crystal system,	hexagonal	monoclinic	monoclinic
space group	*P*65	*P*2_1_/*c*	*C*c
unit cell dimensions			
*a* [Å]	7.5160 (11)	6.7180 (13)	12.044 (2)
*b* [Å]	7.5160 (11)	17.305 (4)	11.961 (2)
*c* [Å]	32.005 (6)	11.455 (2)	20.724 (4)
*α* [º]	90.00	90.00	90.00
*β* [º]	90.00	97.33 (3)	97.47 (3)
*γ* [º]	120.00	90.00	90.00
volume [Å^3^]	1565.7 (5)	1320.8 (5)	2960.1 (10)
*Z*	6	4	4
*ρ*_calcd_ [g cm^−3^]	1.293	1.314	1.316
μ [mm^−1^]	0.087	0.095	0.098
*F* (000)	648	552	1248
crystal size [mm^3^]	0.05 × 0.10 × 0.20	0.10 × 0.10 × 0.20	0.10 × 0.10 × 0.20
*θ* range [º] for data collection	3.13 to 25.31	2.14 to 25.27	1.98 to 25.27
index ranges	0 ≤ h ≤ 7	0 ≤ h ≤ 8	0 ≤ h ≤ 14
	0 ≤ k ≤ 7	0 ≤ k ≤ 20	0 ≤ k ≤ 14
	−38 ≤ l ≤ 38	−13 ≤ l ≤ 13	−24 ≤ l ≤ 24
reflections collected	2,331	2,603	2,956
independent reflections	1898 [ *R*_int_ = 0.097]	2392 [ *R*_int_ = 0.023]	2818 [ *R*_int_ = 0.084]
max. and min. transmission	0.9957/0.9829	0.9905/0.9812	0.9903/0.9807
data/restraints/parameters	1898/1/136	2392/0/173	2818/3/387
goodness-of-fit on *F*^2^	1.005	1.000	1.009
final *R* indices [*I* > 2*σ* (*I*)]; *R*_1_, *wR*_2_	0.0638, 0.1667	0.0552, 0.1488	0.0651, 0.1526
*R*_1_, *wR*_2_ (all data)	0.0992, 0.1901	0.0886, 0.1704	0.0969, 0.1716
largest diff. peak and hole [e·Å^−3^]	0.153 and −0.160	0.239 and −0.188	0.224 and −0.273

**Table 2:** Selected molecular structure parameters of AA.

Parameters	AA	
Bond lengths (Å)	Experimental	B3LYP/6-31G (d)
O1-C7	1.229 (6)	1.228
N1-C7	1.379 (6)	1.400
N1-C6	1.418 (6)	1.417
N1-N2	1.431 (5)	1.420
N2-C9	1.402 (6)	1.421
N2-C10	1.459 (6)	1.475
N3-C8	1.365 (7)	1.394
**Bond angles ** **(°)**		
C7-N1-C6	126.9 (4)	125.11
C6-N1-N2	119.3 (4)	119.28
C9-N2-C10	117.9 (4)	115.26
N1-N2-C10	110.3 (4)	111.49
C1-C6-N1	122.7 (5)	120.76
C5-C6-N1	117.2 (4)	119.23
O-C7-N1	125.1 (4)	126.98
O-C7-C8	129.2 (5)	127.79
N1-C7-C8	105.7 (4)	105.17
C9-C8-N3	129.9 (5)	132.14
N3-C8-C7	121.4 (5)	119.44
N2-C9-C11	119.3 (4)	119.85

**Table 3:** Selected molecular structure parameters of FAA.

Parameters	FAA	
Bond lengths (Å)	Experimental	B3LYP/6-31G (d)
O1-C9	1.248 (5)	1.226
N1-C9	1.384 (5)	1.405
N1-C6	1.424 (5)	1.420
N1-N2	1.412 (5)	1.413
N2-C7	1.359 (5)	1.405
N2-C10	1.463 (5)	1.474
N3-C8	1.419 (5)	1.396
N3-C12	1.305 (6)	1.382
O2-C12	1.228 (5)	1.217
C9-N1-C6	124.3 (3)	124.92
C6-N1-N2	118.3 (3)	119.36
C7-N2-C10	123.0 (4)	116.78
N1-N2-C10	117.4 (4)	112.61
C1-C6-N1	119.4 (4)	119.25
C5-C6-N1	120.4 (4)	120.57
O1-C9-N1	123.6 (4)	125.40
O1-C9-C8	131.7 (4)	130.29
N1-C9-C8	104.7 (4)	104.27
C7-C8-N3	127.8 (4)	127.35
N3-C8-C9	122.7 (4)	124.17
N2-C7-C11	120.4 (4)	120.17
C12-N3-C8	122.2 (4)	127.67
O2-C12-N3	124.7 (5)	121.89

**Table 4:** Selected molecular structure parameters of MMAA.

Parameters	MMAA	
Bond lengths (Å)	Experimental	B3LYP/6-31G (d)
O3-C4	1.235 (3)	1.228
N2-C4	1.396 (3)	1.400
N2-C8	1.422 (3)	1.418
N2-N3	1.408 (3)	1.415
N3-C5	1.384 (3)	1.411
N3-C6	1.476 (3)	1.474
N1-C3	1.407 (3)	1.397
N1-C2	1.346 (3)	1.374
O2-C2	1.208 (3)	1.217
O1-C2	1.342 (3)	1.357
O1-C1	1.428 (4)	1.433
**Bond angles ** **(°)**		
C4-N2-C8	125.2 (2)	125.32
C8-N2-N3	118.9 (2)	119.58
C5-N3-C6	119.8 (2)	116.43
N2-N3-C6	114.1 (2)	112.43
C13-C8-N2	120.7 (3)	120.77
C9-C8-N2	119.2 (2)	119.11
O3-C4-N2	123.9 (2)	127.11
O3-C4-C3	130.6 (2)	127.96
N2-C4-C3	105.5 (2)	104.86
C5-C3-N1	129.6 (2)	133.63
N1-C3-C4	121.5 (2)	117.07
N3-C5-C7	120.3 (2)	118.98
C2-N1-C3	124.0 (2)	125.31
O2-C2-N1	126.1 (2)	126.26
O1-C2-N1	109.5 (2)	109.11
O2-C2-O1	124.4 (2)	124.62
C2-O1-C1	116.8 (2)	114.74

**Table 5:** Selected molecular structure parameters of MCAA.

Parameters	MCAA	
Bond lengths (Å)	Experimental	B3LYP/6-31G(d)
O3-C6	1.240 (8)	1.225
N3-C6	1.386 (9)	1.415
N3-C9	1.420 (7)	1.419
N2-N3	1.420 (7)	1.414
N2-C5	1.373 (8)	1.396
N2-C8	1.459 (8)	1.472
N1-C4	1.412 (8)	1.409
N1-C2	1.367 (10)	1.378
O2-C2	1.215 (8)	1.218
O1-C2	1.351 (9)	1.361
O1-C1	1.411 (10)	1.434
N1-C3	1.456 (9)	1.468
**Bond angles ** **(°)**		
C6-N3-C9	126.3 (5)	124.67
C9-N3-N2	120.6 (5)	119.17
C5-N2-C8	123.9 (6)	117.83
N3-N2-C8	118.2 (5)	113.26
C10-C9-N3	120.6 (5)	119.01
C14-C9-N3	117.6 (6)	120.91
O3-C6-N3	123.1 (6)	125.21
O3-C6-C4	131.6 (6)	130.54
N3-C6-C4	105.2 (5)	104.21
C5-C4-N1	125.6 (6)	128.24
N1-C4-C6	125.0 (6)	123.01
N2-C5-C7	120.8 (6)	120.67
C2-N1-C4	120.7 (6)	123.23
O2-C2-N1	125.0 (7)	124.89
O1-C2-N1	111.2 (6)	111.66
O2-C2-O1	123.8 (8)	123.45
C2-O1-C1	114.9 (7)	114.12
C2-N1-C3	120.0 (6)	118.43
C4-N1-C3	118.5 (6)	117.53

Regarding the crystal structure of AA (Figure 2, Table 6), there is one intermolecular N3-H3•••O hydrogen bond, between the carbonyl oxygen atoms of the pyrazole rings and the hydrogen atoms of the amide groups, and two C-H•••π interactions (C11-H11•••Cg1, and C11-H13•••Cg1), between the methyl hydrogen of the pyrazole ring and the center of phenyl ring, to form a three-dimensional network. The dihedral angle between the pyrazole ring and the phenyl ring is 42.39°.

**Table 6:** Hydrogen bonding geometries (Å, °).

D-H···A	D-H	H···A	D···A	D–H···A	Symmetry codes
**AA**					
N3-H3A···O	0.86	2.23	3.039 (6)	156.0	x − y, −1 + x, −1/6 + z
C11-H11A···Cg1	0.96	3.21	3.476 (6)	98.2	x − y, −1 + x, −1/6 + z
C11-H11C···Cg1	0.96	2.99	3.476 (6)	112.7	x − y,−1 + x, −1/6 + z
**FAA**					
N3-H3A···O1	0.86	2.01	2.864 (5)	172.0	−x, y + 1/2,−z + 3/2
C10-H10B···Cg1	0.96	2.85	3.733 (5)	153.0	−x − 1, y + 3/2, −z + 3/2
C12-H12A···Cg1	0.93	3.03	3.647 (5)	125.0	−x, y+ 3/2, −z + 3/2
**MMAA**					
N1-H1A···O3	0.86	2.01	2.850 (3)	166.0	1 − x, 1 − y, 2 − z
C9-H9A···O3	0.93	2.52	2.960 (3)	109.0	
C1-H1C···Cg1	0.96	3.11	3.686 (4)	120.1	x, 1/2 − y, 1/2 + z
C7-H7B···Cg1	0.96	3.23	3.763 (3)	116.7	2 − x, 1− y, 2− z
**MCAA**					
OW1-HW1B···O3	1.01	1.90	2.850 (9)	156.0	
C17-H17A···O5	0.96	2.34	2.766 (10)	106.0	
OW2-HW2B···O3	0.85	2.51	2.860 (7)	106.0	
OW2-HW2A···O3	0.85	2.55	2.860 (7)	102.0	
OW2-HW2A···O6	0.85	2.41	2.871 (8)	114.0	
C3-H3A···O2	0.96	2.43	2.806 (10)	103.0	
OW1-HW1A···O6#1	0.96	1.90	2.823 (9)	159.0	1/2 + x, 1/2 + y, z
C8-H8C···OW1#2	0.96	2.49	3.056 (10)	118.0	−1/2 + x, 1/2 + y, z
C10-H10A···OW2#2	0.93	2.52	3.358 (9)	151.0	−1/2 + x, 1/2 + y, z
C22-H22A···O5#2	0.96	2.56	3.510 (8)	171.0	−1/2 + x, 1/2 + y, z
C28-H28A···OW2#2	0.93	2.54	3.384 (9)	151.0	−1/2 + x, 1/2 + y, z
C3-H3B···Cg1#3	0.96	3.20	4.038 (9)	147.0	x, y, z
C8-H8B···Cg1#1	0.96	2.80	3.737 (8)	165.3	1/2 + x, 1/2 + y, z
C14-H14A···Cg2#1	0.93	3.11	3.582 (7)	113.0	1/2 + x,1/2 + y, z
C17-H17B···Cg3#3	0.96	3.36	4.020 (9)	127.4	x, y, z
C22-H22B···Cg3#4	0.96	2.90	3.814 (8)	160.5	−1/2 + x,−1/2 + y, z
C24-H24A···Cg4#4	0.93	3.12	3.539 (8)	109.4	−1/2 + x, −1/2 + y, z

The molecular conformation of MMAA (Figure 4, Table 6) is stabilized *via* the intermolecular N1-H1•••O3 hydrogen bonds, between the carbonyl oxygen atoms of the pyrazole rings and the hydrogen atoms of the amide groups to form a ten-member-ring, and two C-H•••π interactions, between the methoxy hydrogen and the phenyl ring (C1-H1•••Cg1, C7-H7•••Cg1), to form dimers.

**Figure 6:**
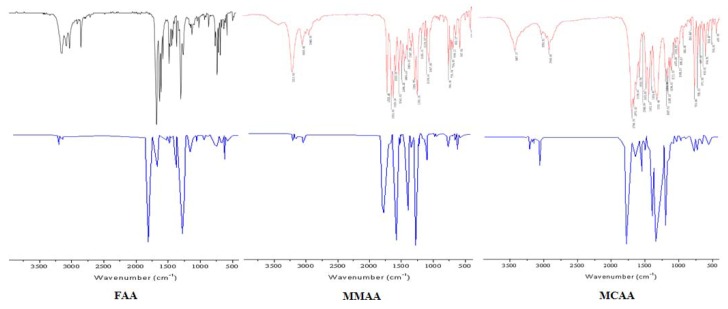
Experimental (**above**) and Theoretical (**below**) FT-IR of the compounds.

In the crystal of MCAA (Figure 5, Table 6), water molecules are involved in the intermolecular hydrogen bonds between two molecules of MCAA. The water molecules were confirmed in the IR (Figure 6). There are three C-H•••π interactions (C3-H3•••Cg1, C8-H8•••Cg1, C14-H14•••Cg2) to reinforce the crystal packing. The crystal structure of FAA has been reported by us [13].

### 2.2. Vibration Spectra

Vibration spectroscopy is used extensively in organic chemistry for the identification of functional groups of organic compounds, the study of molecular conformations, reaction kinetics, *etc*. The vibration spectral data obtained from the solid phase FT-IR spectra are assigned based on the results of the normal coordinate calculations. The experimental and the simulated infrared spectra, where the intensity (km/mol) is plotted against the vibration frequencies, are shown in Figure 6. The resulting vibration wave numbers for the optimized geometry and the proposed assignments are given in Table 7. As seen from Table 7, the observed and the calculated spectra are in good agreement with each other.

**Table 7:** Experimental and Theoretical FT-IR and assignments for the compounds (cm^−1^).

FAA			MMAA			MCAA		
Exp.	B3LYP/6-31G *	Vibrational assignments	Exp.	B3LYP/6-31G *	Vibrational assignments	Exp.	B3LYP/6-31G *	Vibrational assignments
3190ms	3187	*ν*_N-H_	3213s	3210	*ν*_N-H_	3056w	3055	*ν*_=C-H_
3049ms	3044	*ν*_=C-H_	3053m	3057	*ν*_=C-H_	2948m		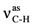
2925ms		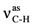	2948w		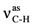	1700vs	1690	*ν*_C=O_
2878ms		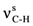	1725vs	1727	*ν*_C=O_	1676vs	1659	*ν*_C=O_
1689vs	1690	*ν*_C=O_	1659vs	1659	*ν*_C=O_	1639s	1643	*ν*_C=C_
1636vs	1643	*ν*_C=O_	1629vs		*ν*_C=C_	1593ms		ν_C=C_
1545s	1544	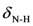	1593s		ν_C=C_	1544w	1546	
1490s	1480	ν_C=C_	1541s	1538	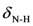	1493s	1498	ν_C=C_
1386ms	1395	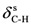	1494s	1492	ν_C=C_	1452vs	1455	ν_C=C_
1216ms	1211	ν_C-N_	1456ms	1449	ν_C=C_	1331vs	1340	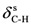
1140m	1129	ν_C-N_	1347m	1346	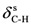	1205ms	1209	ν_C-N_
1106w	1113	ν_C-N_	1290s		ν_C-N_	1187ms	1188	ν_C-N_
1020w	1017	ν_C-N_	1253vs		ν_C-O_	1165ms	1169	ν_C-O_
856w	850		1185w	1188	ν_C-N_	1137ms	1141	ν_C-N_
768ms	774	*γ*_=C-H_	1139w	1141	ν_C-N_	1111ms	1108	ν_C-N_
698ms	708	γ_N-H_	1109w	1117	ν_C-N_	1073w	1072	ν_C-O_
666w	659	*γ*_=C-H_	1067s	1065	ν_C-O_	1051w	1045	ν_C-N_
638w	632		765s	758	*γ*_=C-H_	996m	991	
			735m	740		769s	773	*γ*_=C-H_
			714m	709	γ_N-H_	725m	728	
			694m	703	*γ*_=C-H_			
			652w	661				

vs, very strong; s, strong; ms, medium strong; m, medium; w, weak; vw, very weak; ipb, in plane bending; opb, out plane bending.

FAA or MMAA have one N-H bond. The characteristic IR band of the synthesized FAA or MMAA appears the peak in the 3190 and 3213 cm^−1^ regions due to the (N-H) stretching vibrations. This is interpreted as a result of their conjugated resonance with the pyrazole ring, besides the carbonyl group is connected to the imine group. The calculated (N-H) stretching vibrations are observed at 3187 and 3210 cm^−1^, respectively.

There are aromatic moieties in the molecules of FAA, MMAA, and MCAA. The stretching bands of C-H (Ar-H) appear at 3049, 3053, and 3056 cm^−1^, respectively. These values have been calculated as 3044, 3057, and 3055 cm^−1^, respectively.

FAA, MMAA, and MCAA have two kinds of carbonyl (C=O) groups. The very strong stretching bands of amide carbonyl appear at 1689, 1725, and 1700 cm^−1^, respectively, while they are calculated at 1690, 1727, and 1690 cm^−1^; The very strong stretching bands of pyrazole carbonyl appear at 1636, 1659, and 1676 cm^−1^, respectively, apparently decreasing in frequencies compared with the carbonyl absorption of AA (1679 cm^−1^), while they are calculated at 1643, 1659, and 1659 cm^−1^. The assignment of the experimental frequencies is based on the observed band frequencies in the infrared spectra (Table 7).

### 2.3. Theoretical Structures

The optimized parameters (bond lengths and bond angles) of AA, FAA, MMAA, and MCAA were obtained by using B3LYP/6-31G (d) method and listed in Table 2, Table 3, Table 4, Table 5 to compare with the X-ray experimental data.

As seen from Table 2, the biggest difference between the X-ray and calculated values of the bond lengths of AA is at N3-C8. The calculated value is 0.0291 Å longer than the X-ray value. The biggest difference between the X-ray and calculated values of the bond angles of AA is at C9-N2-C10. The calculated value is 2.2356° smaller than the X-ray value. It is because there are intermolecular N3-H3•••O hydrogen bond and the C-H•••π interactions (Figure 2), there are such differences between the X-ray and calculated values.

As seen from Table 3, for the same reason, the biggest differences between the X-ray and the calculated values of the bond length and angles of FAA is at N3-C12 and at C7-N2-C10, respectively. The differences are 0.0773 Å and 6.2166°, respectively.

As seen from Table 4, the biggest difference between the X-ray and the calculated values of the bond lengths and angles of MMAA is at N1-C2 and at N1-C3-C4, respectively. The differences are 0.0278 Å and 4.4298°, respectively. The reasons could be that the carbonyl oxygen atom of the pyrazole ring is involved not only in the intermolecular hydrogen bond (N1-H1•••O3) but also in the intramolecular hydrogen bond (C9-H9···O3), and that there are C-H•••π interactions between the molecules.

**Figure 7:**
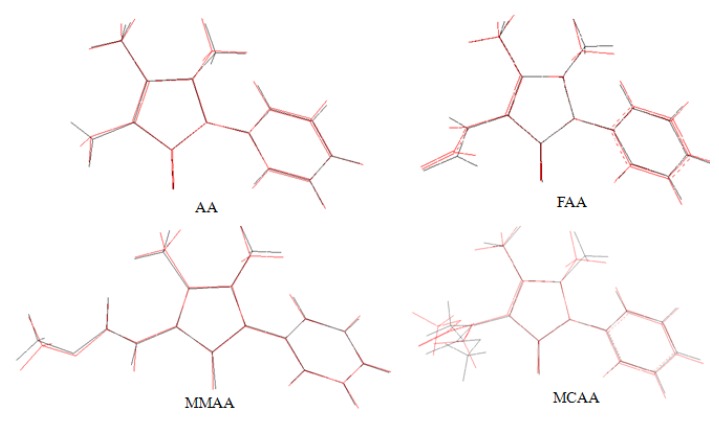
Atom-by-atom superimposition of the calculated structure (red) over the X-ray structure (black) for the compounds.

As seen from Table 5, the biggest difference between the X-ray and the calculated values of the bond lengths and angles of MCAA is at N3-C6 and at C5-N2-C8, respectively. The differences are 0.0285 Å and 6.0658°, respectively. When the X-ray structures of the compounds are compared with their optimized counterparts (Figure 7), conformational discrepancies are observed between them.

The most notable discrepancies exist in the orientation of the methyl groups, which are attached to N(2) and C(3) of the pyrazole ring in the compounds. For AA, FAA, MMAA, and MCAA, the orientation of the methyl groups were defined by the torsion angles in X-ray data [134.5 (4)°, −148.5 (4)°, 139.3 (2)°, and −151.4 (6)°] and [177.0 (5)°, 178.8 (5)°, −179.9 (3)°, and 175.0 (7)°], respectively. They were calculated as 133.06°, −135.19°, 131.28°, and 134.18°, and −179.8981°, 178.92°, 179.43°, and −177.90°, respectively.

The molecular structures of the compounds are nonplanar. According to the X-ray study, the dihedral angles between the pyrazole ring and the benzene ring are 42.39°, 50.03°, 36.99°, and 41.17° for AA, FAA, MMAA, and MCAA, respectively, whereas the dihedral angles have been calculated as 45.22°, 74.47°, 25.43°, and 38.91°, respectively.

We noted that the experimental results correspond to the solid phase of the compounds and that the theoretical calculations are for the gas phase. In the solid state, there are intermolecular hydrogen bonds between molecules, and the experimental results are related to molecular packing, while isolated molecules are considered in the theoretical calculations. In spite of these small differences, calculated geometric parameters represent a good approximation and they are the basis for calculating other parameters, such as frontier orbitals and energy, and molecular electrostatic potential, as we describe later.

### 2.4. Frontier Molecular Orbital Analysis

**Figure 8:**
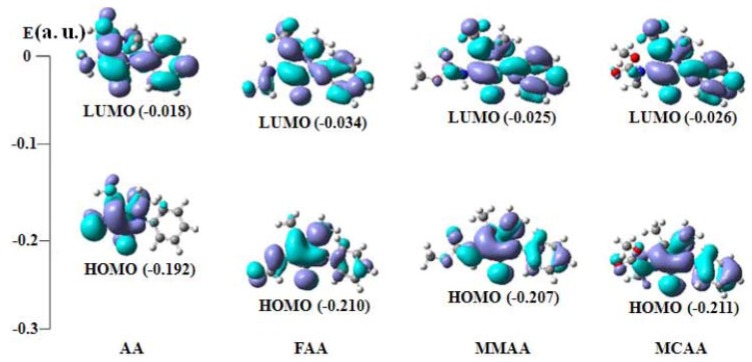
Frontier molecular orbital of the compounds.

Molecular orbital and their properties, like energy, are very useful for physicists and chemists and their frontier electron density used for predicting the most reactive position in p-electron systems and also explained several types of reaction in conjugated system [14]. Moreover, eigenvalues of the lowest unoccupied molecular orbital (LUMO) and the highest occupied molecular orbital (HOMO) and their energy gap reflect the chemical activity of the molecule. Recently the energy gap between HOMO and LUMO has been used to prove the bioactivity from intramolecular charge transfer (ICT) [15,16]. The HOMO-LUMO energy gaps for the four compounds were calculated by B3LYP/6-31G (d). From the HOMO-LUMO orbital picture (Figure 8), it is found that the filled p-orbital (HOMO) is mostly located on the pyrazole ring and -N(H) group of the compounds, while the unfilled antip-orbital (LUMO) is on the benzene ring. When electron transitions take place, electrons are mainly transferred from the pyrazole ring and -N(H) group to the phenyl ring. Therefore, introduction of an electron withdrawing group into the -N(H) group will reduce the energy of the HOMO. It can be seen from Figure 8 and Table 8 that the HOMO energy of AA is highest (−0.192 a. u.), and that the gap is the smallest (0.174 a. u.). It implies that the electronic transfer in AA is easier.

**Table 8:** Frontier orbital energy (a.u.).

Compound	E(HOMO)	E (LUMO)	∆E (LUMO−HOMO)
AA	−0.192 (54a)	−0.018 (55a)	0.174
FAA	−0.210 (61a)	−0.034 (62a)	0.176
MMAA	−0.207 (69a)	−0.025 (70a)	0.182
MCAA	−0.211 (73a)	−0.026 (74a)	0.185

### 2.5. Molecular Electrostatic Potential

Molecular electrostatic potential (MEP) is related to the electronic density and is a very useful descriptor in understanding sites for electrophilic attack and nucleophilic reactions as well as hydrogen bonding interactions [17,18,19]. The electrostatic potential V(r) are also well suited for analyzing processes based on the “recognition” of one molecule by another, as in drug-receptor and enzyme-substrate interactions, because it is through their potentials that the two species first “see” each other [20,21]. Being a real physical property, V(r)s can be determined experimentally by diffraction or by computational methods [22].

Many researchers have used graphic models, especially MEP, as a tool in conformational analysis [23]. The fundamental application of this study is the analysis of noncovalent interactions [24,25,26,27], mainly by investigating the electronic distribution in the molecule. Thus, this methodology was used to evaluate the electronic distribution around molecular surface for the four compounds.

**Figure 9:**
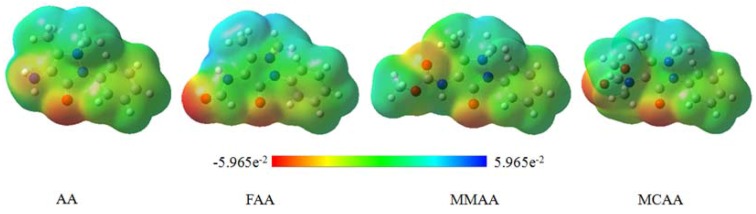
Molecular electrostatic potential map of the compounds.

To visually consider the most probable sites of the molecules for an interaction with electrophilic and nucleophilic species, MEP was calculated at the B3LYP/6-31G (d) optimized geometry. While electrophilic reactivities are visualized by red color which indicates the negative regions of the molecule, the nucleophilic reactivities are colored in blue, indicating the positive regions of the molecule, as shown in Figure 9. The nitro and carbonyl oxygen atoms are surrounded by a greater negative charge surface, becoming these sites potentially more favorable for electrophilic attack. As can be seen from the results, the MEP map confirms the existence of intra- and intermolecular interactions observed in the solid state.

**Figure 10:**
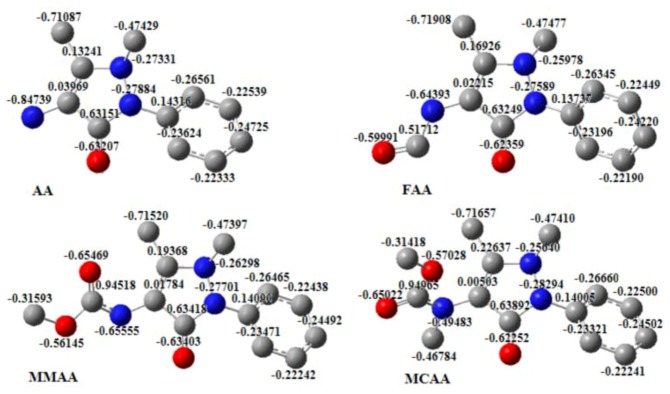
Electronic density of the atoms.

In the MEP of AA, the main negative center includes the nitrogen atom attacked at C(4) of pyrazole ring and the pyrazole carbonyl group, which should be responsible for the interaction with the active drug-receptor sites [28]. It is clear in the MEPs that around the nitrogen atoms attached at C(4) of the pyrazole ring, FAA, MMAA and MCAA show an electronic density lower than those of AA. That is, there is a larger electronic concentration in the active sites of AA. It could be the reason for the preferential COX_2_-drug binding and in agreement with the activity observed in AA. A significant change in the molecular structure of the compounds is the presence of a different substituent attached to C(4). For AA, the substituent at C(4) is -NH_2_, while for FAA, MMAA, and MCAA, the substituents are -NHCHO, -NHCOOCH_3_, and -NH(CH_3_)COOCH_3_, respectively. The amide carbonyl substituents should increase the electronic delocalization in the molecules. The electronic density of the atoms is shown in Figure 10.

## 3. Experimental

### 3.1. General

Melting points were measured on an X-4 microscope electro-thermal apparatus (Taike, Nanjing, China) and were uncorrected. ^1^H-NMR spectra were recorded on a Bruker spectrometer at 500 MHz using CDCl_3_ as the solvent, with tetramethylsilane as an internal standard. IR spectra were recorded in KBr disk using a Nicolet 380 FT-IR spectrophotometer. Elemental analyses were performed with a Flash EA-1112 elemental analyzer. Crystal data were collected on a Nonius CAD-4 diffractometer by using MoKα (0.71073 Å) irradiation.

### 3.2. Synthesis

AA was a commercial product (Yacoo Corperation, Suzhou, China). FAA, MMAA, and MCAA were synthesized as follows: AA (10 mmol), formic acid (60 mmol), and ZnO (5 mmol) were placed in a 25 mL three-necked round bottom flask. The reaction was started by stirring and heating to 70 °C. The reaction was monitored by TLC (eluent: ether/acetone, 1:2 v/v). CH_2_Cl_2_ (20 mL) was added after the reaction was completed. ZnO was removed by filtration. The filtrate was washed with H_2_O (2 × 10 mL) followed dry saturated aqueous sodium bicarbonate (2 × 10 mL), and dried over anhydrous Na_2_SO_4_. Yellow powder of FAA was got Yield: 12%. M.p. 192–194 °C; Elemental analysis: Anal. Calcd for C_12_H_13_N_3_O_2_: C 62.33, H 5.67, N 18.17; found C 62.56, H 5.64, N 18.11; ^1^H-NMR (CDCl_3_) *δ*: 9.09 (s, 1H, CHO), 8.23 (s, 1H, NH), 7.48–7.31 (m, 5H, Ar-H), 3.09 (s, 3H, CH_3_), 2.25 (s, 3H, CH_3_); IR (KBr, cm^−1^) υ: 3190 (*ν*_N-H_), 3049 (*ν*_=C-H_), 2925 (*ν*_C-H_), 2878 (*ν*_C-H_), 1689 (*ν*_C=O_), 1636 (*ν*_C=O_), 1545 (d_N-H_), 1490 (*ν*_C=C_), 1386 (d_C-H_), 1216 (*ν*_C-N_), 1140 (*ν*_C-N_), 768 (γ_=C-H_), 698 (γ_N-H_).

AA (10 mmol) was added to a mixture of Me_2_CO_3_ (18 mL), 18-crown-6 (0.1 mmol), and NaH (25 mmol). The reaction mixture was heated in an oil bath to 50 °C. The reaction was monitored by TLC (eluent: acetone:chloroform, 2:1 v/v). Me_2_CO_3_ was evaporated under reduced pressure after the reaction was completed. The residue was mixed with 150 mL water. The insoluble solid was removed by filtration. The liquid was extracted with CH_2_Cl_2_. The combined organic extracts were dried (Na_2_SO_4_) and evaporated under reduced pressure to give MMAA as a light yellow powder. Yield: 73%. M.p. 180–182 °C; Elemental analysis: Anal. Calcd for C_13_H_15_N_3_O_3_: C 59.76, H 5.79, N 16.08; found C 59.98, H 5.82, N 16.02; ^1^H-NMR (CDCl_3_) *δ*: 7.46–7.27 (m, 5H, Ar-H), 3.71 (s, 3H, OCH_3_), 3.05 (s, 3H, CH_3_), 2.23 (s, 3H, CH_3_); IR (KBr, cm^−1^) υ: 3213 (*ν*_N-H_), 3053 (*ν*_=C-H_), 2948 (*ν*_C-H_), 1725 (*ν*_C=O_), 1659 (*ν*_C=O_), 1629 (*ν*_C=C_), 1593 (*ν*_C=C_), 1541 (d_N-H_), 1494 (*ν*_C=C_), 1456 (*ν*_C=C_), 1347 (d_C-H_), 1290 (*ν*_C-N_), 1253 (*ν*_C-O_), 1139 (*ν*_C-N_), 1067 (*ν*_C-O_), 765 (γ_=C-H_), 714 (γ_N-H_).

K_2_CO_3_ (30 mmol) and dimethyl carbonate (36 mL) was added to a mixture of AA (10 mmol) and 18-crown ether-6 (0.6 mmol). The mixture was stirred and heated in an oil bath at 90 °C. The reaction was monitored by TLC (eluent: acetone/chloroform, 2:1 v/v). The excess dimethyl carbonate was removed in vacuum after the reaction was completed. CH_2_Cl_2_ (20 mL) was added to the residue. The insoluble solid was filtrated. After removal of the solvent, the brown residue was recrystallized from the mixed solvent of ether and petroleum ether. A brown solid of MCAA was obtained. Yield: 80%. M.p. 120–121 °C; Elemental analysis: Anal. Calcd for C_14_H_17_N_3_O_3_: C 61.08, H 6.22, N 15.26; found C 60.88, H 6.19, N 15.32; ^1^H-NMR (CDCl_3_) *δ*: 7.48–7.26 (m, 5H, Ar-H), 3.71 (s, 3H, OCH_3_), 3.20 (s, 3H, CH_3_), 3.09 (s, 3H, CH_3_), 2.15 (s, 3H, CH_3_); IR (KBr, cm^−1^) υ: 3056 (*ν*_=C-H_), 2948 (*ν*_C-H_), 1700 (*ν*_C=O_), 1676 (*ν*_C=O_), 1639 (*ν*_C=C_), 1593 (*ν*_C=C_), 1493 (*ν*_C=C_), 1452 (*ν*_C=C_), 1331 (d_C-H_), 1165 (*ν*_C-O_), 1137 (*ν*_C-N_), 1073 (*ν*_C-O_), 769 (γ_=C-H_).

### 3.3. Crystallography

Single crystals of AA, MMAA, and MCAA were prepared by recrystallization from acetonitrile, acetone, and diethyl ether, respectively. The X-ray diffraction data were collected on an automated Enraf-Nonius CAD-4 diffractometer (Mo-Ka radiation, 0/20 scanning technique). The positions and thermal parameters of the non-hydrogen atoms were refined anisotropically. H atoms were positioned geometrically and refined as riding groups, with N-H = 0.86Å (for NH), C-H = 0.93, 0.93 and 0.96Å (for aromatic, aldehydic and methyl H), respectively, and constrained to ride on their parent atoms, with Uiso (H) = xUeq (C), where x = 1.2 for aromatic H, and x = 1.5 for other H. The positions of the hydrogen atoms were located according to the difference of electron density. All calculations were carried out with the use of the SHELXL-97 program package. Details of the parameters are given in Table 1. CCDC-660447, 801822, 801827, 801826 contains the supplementary crystallographic data for this paper. These data can be obtained free of charge at www.ccdc.cam.ac.uk/conts/retrieving.html [or from the Cambridge Crystallographic Data Centre (CCDC), 12 Union Road, Cambridge CB2 1EZ, UK; Fax: +44(0) 1222-336033; email: deposit@ccdc.cam.ac.uk].

### 3.4. Theoretical Calculation

The molecular structures of the compounds in the ground state (*in vacuo*) were optimized using DFT (B3LYP) method with the 6-31G (d) basis set with the Gaussian 03 software package. The initial configurations for calculation were constructed according to the X-ray data. Frequency calculations at the same levels of theory revealed no imaginary frequencies, indicate that the B3LYP/6-31G (d) method was the optimal one in our system.

## 4. Conclusions

In this study, three AA derivatives (FAA, MMAA, and MCAA) have been synthesized and characterized by elemental analysis, FT-IR, and ^1^H-NMR spectroscopy. AA and the three derivatives were characterized by single-crystal X-ray diffraction techniques. The theoretical calculations of AA and the derivatives have been performed by using the density functional theory (DFT) method with the 6-31G(d) basis set. Although differences were observed in the geometric parameters, the general agreement is in a good range and the theoretical calculations support the solid-state structures. The experimental vibration frequencies are in a good agreement with the results of the B3LYP method. The calculated MEP map verifies the solid-state interactions.
